# The variations of VP1 protein might be associated with nervous system symptoms caused by enterovirus 71 infection

**DOI:** 10.1186/1471-2334-14-243

**Published:** 2014-05-07

**Authors:** Bao Zhang, Xianbo Wu, Keyong Huang, Ling Li, Li Zheng, Chengsong Wan, Ming-Liang He, Wei Zhao

**Affiliations:** 1 School of Public Health and Tropical Medicine, Southern Medical University, NO.1023 Shatai Road, Guangzhou 510515, P.R. China; 2 Stanley Ho Center for Emerging Infectious Diseases, and Li Ka Shing Institute of Health Sciences, Faculty of Medicine, and Shenzhen Research Institute of The Chinese University of Hong Kong, Hong Kong, China

**Keywords:** EV71, Neurovirulence, VP1, Variation

## Abstract

**Background:**

The VP1 protein of enterovirus 71 (EV71) is an important immunodominant protein which is responsible for host-receptor binding. Nevertheless, the relationship between VP1 and neurovirulence is still poorly understood. In this study, we investigated the relationship between mutation of VP1 and neurovirulent phenotype of EV71 infection.

**Methods:**

One hundred and eighty-seven strains from Genbank were included, with a clear clinical background. They were divided into two groups, one with nervous system symptoms and one with no nervous system symptoms. After alignment, the significance of amino acid variation was determined by using the χ^2^ test and a phylogenetic tree was constructed with MEGA software (version 5.1).

**Results:**

We showed no significant difference in neurovirulence between genotype B and C. Interestingly, we found that variations of E145G/Q, E164D/K and T292N/K were associated with nervous system infection in genotype B. In the case of genotype C, the N31D mutation increased the risk for nervous complications, whereas I262V mutation decreased the risk of nervous complications. We used a 3D model of VP1 to demonstrate the potential molecular basis for EV71 nervous system tropism.

**Conclusions:**

Distinct variations are shown to be associated with neurovirulent phenotype in the different genotype. Detection of variation in genotypes and subtypes may be important for the prediction of clinical outcomes.

## Background

Enterovirus 71 (EV71) is a member of human enterovirus species, which is one of the most common causative agents for herpangina and hand, foot and mouth disease (HFMD) [1,2]. EV71 infection can also result in acute cardiogenic shock, pulmonary edema, and severe neurological diseases such as encephalitis and meningitis, the major causes for mortality among infants and young children [3,4], as well as in adults [5]. Recently, outbreaks of EV71 infection have been frequently reported throughout the world, including China [6], South Korea [7], Taiwan [8], Malaysia [9] and Singapore [10].

Although molecular epidemiological studies have been extensively conducted by different groups [6,9,11-16], little has been done to reveal the correlation of specific viral genotypes or residue variations and nervous system tropism[17,18]. The mechanism of the neurological complications of enterovirus infection is poorly understood. Neurological virulence is one of the most severe complications responsible for death [19]. It has been reported that patients with central nervous system symptoms accounted for 52.9% (92/174) of EV71 infection [20]. Cordey et al. have demonstrated that an L97R alteration of VP1 protein enhances the neuronal tropism of EV71[17]. Some adaptations of VP1, 5′ NCR, protease 2A determine viral virulence [18]. These results suggested that the sequence variations may contribute to neural infection and neurological complications.

VP1 is one of structural proteins of EV71 virus, which is involved in forming the pentameric icosahedral structure and important for virus to bind receptors of host cells. In this study, we analyzed the correlation between VP1 variations and the neurological virulence caused by EV71 infection.

## Methods

### Nucleotide sequences screening

We took the advantage of BLASTN tool to search VP1 gene of EV71, and cross-validated in the database of EXPASY. The sequences were doubly checked by going through 324 papers one by one. The complete VP1 sequences of 187 EV71 strains (Additional file 1: Table S1) were downloaded from Genbank as of Sep 30, 2012. Seventy-six strains isolated from patients with nervous system symptoms (e.g., encephalitis, aseptic meningitis, and poliomyelitis-like paralysis), were referred to as nervous system EV71 strains (NS-EV71), and the other 111 strains from only HFMD and mild HFMD were considered as non-nervous system EV71 strains (nNS-EV71).

### Alignment and phylogenetic tree construction

Sequence alignment and phylogenetic tree was constructed with MEGA software (version 5.1) using the neighbor-joining method. The robustness of the constructed phylogeny was estimated by bootstrap analysis with 1,000 pseudo-replicate data sets.

### 3D reconstruction of VP1 protein

The crystal structure of the VP1-VP4 protein complex of EV 71 (PDB ID: 3VBS) was obtained from NCBI database (http://www.ncbi.nlm.nih.gov/Structure/mmdb/mmdbsrv.cgi?uid=97658). The 3D structure of VP1 protein was modeled by using free software Cn3D (version 4.3.1, http://www.ncbi.nlm.nih.gov/Structure/CN3D/cn3d.shtml).

### Statistics

The significance of the amino acid variations associated with neurovirulent phenotype was detected using the χ^2^ test by SPSS 13.0 software. A *p* value less than 0.05 was considered as statistically significance.

## Results

### Phylogenetic analysis of VP1

**Figure 1:**
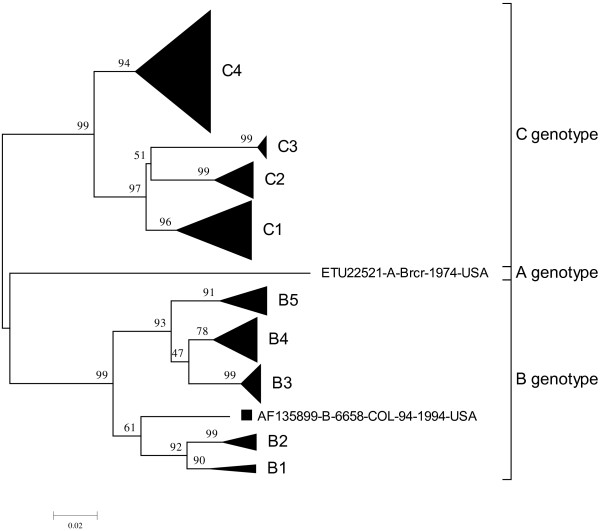
**Phylogenetic dendrogram constructed by using complete VP1 gene sequence of 187 EV71 strains.** Genotypes and subtypes are shown on the right, and bootstrap values (percentage of 1,000 pseudoreplicates) are shown at the nodes of the major clades. All subtypes branches are combined except for strain AF135899.

One hundred and eighty-seven strains were carefully screened from Genbank and Pubmed, and divided into two groups. NS-EV71 group contained 76 strains, and nNS-EV71 had 111 strains. A phylogenetic tree was constructed by using 891 nucleotides of VP1 sequences of 187 EV71 strains (Figure 1 and Additional file 1: Figure S1A/B for genotype B, C). These strains were divided into three groups: A, B (B1–B5) and C (C1–C4) genotypes. The strain AF135899 was included in B genotype, but did not belong to B1-B5. Except subtypes B1 and B2, there were no concentrated clusters in NS-EV71 strains in other subtypes of genotype B and C. More importantly, some EV71 subtypes are geographical and time specific, such as, C4 in China and Taiwan, and B4-5 mainly in Malaysia (Additional file 1: Figure S1A/B).

### Genotype/subtype analysis in EV71

The genotype or subtype numbers of NS-EV71 and nNS-EV71 cases and their distribution worldwide are listed in Additional file 1: Table S2. Based on SPSS analysis, genotype B or C was not associated with nervous system symptoms (χ^2^ = 2.021, *p* = 0.103). However, when further analyzed subtypes, we found that the subtypes B1–B3 were positively correlated with nervous system complications (χ^2^ = 16.565, *p* = 0.002). In the case of subtypes in genotype C, no obvious correlation between subtypes and neurovirulence was detected (χ^2^ = 5.396, *p* = 0.145).

### Relationship between amino acid variations and nervous system symptoms

The amino acid sequences were aligned to find the correlation between amino acid variations and nervous system symptoms. Consensus changes were observed to correlate with specific genotype at position 43, 58, 184 and 240 (Table 1). In genotype B, all subtypes had the consensus amino acids 43E, 58 T, 184 T and 240S; and seven other residues displayed high frequency of substitution in both NS-EV71 and nNS-EV71 cases (Table 2). The amino acid changes of E145G/Q (OR, 3.5; 95% CI, 1.228–9.978), E164D/K (OR, 6.652; 95% CI, 1.315–33.647), and T292N/K (OR, 8; 95% CI, 2.294–27.9) were significantly correlated with nervous system symptoms. The other four amino residues were not significantly associated with neurovirulence.

**Table 1:** Consensus amino acids of VP1 in differential genotypes of EV71

**Genotype**	**VP1 amino acid site**			
	**43**	**58**	**184**	**240**
A	K	A	S	S
B	E	T	T	S
C	K	A	S	T

**Table 2:** Variation of amino acids of VP1 in EV71 genotypes B and C

**Genotype B**						**Genotype C**					
**Site**	**Amino acid**	**NS**	**nNS**	**χ**^**2**^	***P***	**Site**	**Amino acid**	**NS**	**nNS**	**χ**^**2**^	***P***
17	G	1	6	3.364	0.073	22	R	10	8	3.9777	0.137
	S	31	30				H	10	25		
							Q	23	42		
98	K	3	4	0.055	0.567	31	D	9	6	4.118	0.042
	E	29	32				N	34	69		
145	G	12	7	6.175	0.046	98	K	12	12	2.866	0.239
	Q	4	1				V	0	1		
	E	16	28				E	31	62		
164	D	8	2	6.510	0.039	145	G	3	6	2.335	0.671
	K	1	0				Q	4	7		
	E	23	34				A	1	0		
179	I	1	2	0.237	0.545		K	0	1		
	V	31	34				E	35	61		
237	N	8	3	3.47	0.062	170	V	3	0	5.369	0.046
	T	24	33				A	40	75		
292	N	15	2	15.426	0.0004	249	V	21	38	0.037	0.5
	K	1	2				I	22	37		
	T	16	32								
						262	V	6	23	4.119	0.033
							I	37	52		
						289	T	16	37	1.624	0.140
							A	27	38		

Genotype C was distinct from genotype B for the consensus sequence with 43 K, 58A, 164D, 184S and 240 T. Three residue substitutions (N31D, V170A and V262I) were associated with nervous system symptoms in C genotype (Table 2). The OR and 95% CI of N31D were 3.044 and 1.002–9.252, indicating that N31D variant was significantly associated with EV71 neurovirulence. More interestingly, we noted that C4 subtype had a consensus residue 262I. To our surprise, the risk of V262I substitution in subtype C1–3 was negatively associated with neurovirulence (χ^2^ = 6.262, p = 0.012; OR, 0.239; 95% CI, 0.075–0.756). As the occurrence rate of A170V was too low, the role of A170V in clinical presentation needs to be verified in larger samples. There was no amino acid position linked with nervous system symptoms in the C4 subtype (data not shown).

### Locations of specific amino acid variation of VP1 protein

**Figure 2:**
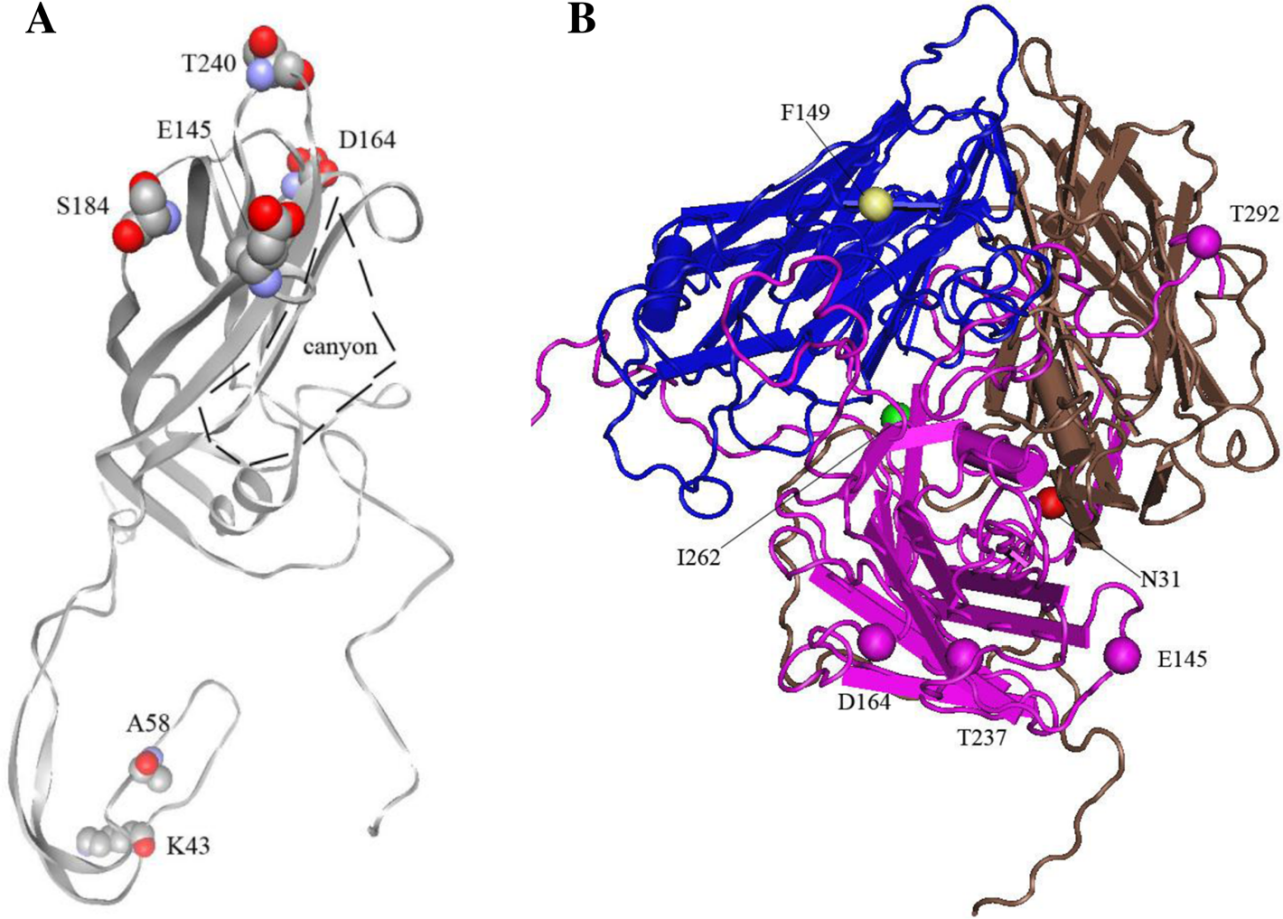
**Location of amino acids in the 3D structure of VP1 protein (A) and the complex (B) of VP1, VP2 and VP3. A** and **B** are based on the PDB 3VBS which belonged to genotype C. the structure of PDB 3VBS was reconstituted with software of Cn3D (version 4.3.1).The consensus residues of K43, A58, S184 and T240 and canyon structure are shown in **A**; the variation sites of N31, E145, D164, I262 and T292 of VP1, F149 of VP2 are shown in **B**.

Aligned with the structure of VP1 (PDB 3VBS), the consensus sequences of genotypes B and C, 184 T/S and 240S/T positioned at the loop of FG and HI regions, protruding the external surface of virus body and the neighbor canyon that is important for the receptor binding; while 43E/K and 58 T/A were located at the N terminus of VP1, buried in the inner of virus body (Figure 2A).

The residue variations of E145G/Q and E164D/K in genotype B were sited at the DE and EF loop, respectively. They located at the protrusion of the canyon surface. However, T292N placed at the C terminus, which was exposed on the virion surface. The alterations of N31D and I262V in genotype C were situated at the N terminus and β-strand I, respectively (Figure 2B).

## Discussion

EV71 is a picornavirus that causes mild HFMD in most cases. However, it is also frequently reported to cause severe nervous system complications. In this study, we found that no significant relationship was exhibited between neurovirulence and genotype or subtype, except subtypes B1 and B2, which is consistent with the previous study [21], although Ooi MH observed that children with B4 genotype were less likely to have CNS infection than those with other genotypes [22].

It has been reported that D164E variation was associated with severe cases of EV71 infection [18]. In the severe cases of EV71 infection patients, the nervous system infection was most frequent. Nervous system tropism for the neural infection might be responsible for the neurovirulence of EV71. The attachment to target cell receptors mediated by VP1 protein was the first and key step for EV71 infection [23]. The residue variations of VP1 protein, which binds receptors on the surface of host cells (e.g., neural cells), may change the specificity of VP1-receptor interaction and therefore play an important role in neurological complications.

So far, four receptors, including P-selectin glycoprotein ligand-1(PSGL-1) [24], scavenger receptor class B member 2 (SCARB2) [25,26], sialylated glycans [27] and heparan sulfate glycosaminoglycan [28], were identified. In our study, we analyzed the complete VP1 protein sequences of 187 strains with detailed clinic information screened from the Genbank database. The results showed that the consensus sequences of genotype B was distinct from genotype C, which consisted of 43E/K, 58 T/A, 184 T/S and 240S/T (Table 1). While 3D structure presented that residue 184 and 240 lay at the protrusion of the pocket that was responsible for recognition and binding to receptors (Figure 2A) [29,30], as indicated that different genotypes of EV71 may bind different receptors. The variation of E145G/Q and E164D/K in genotype B was correlated with neurovirulent phenotype as reported by a previous study [18], but not in genotype C; meanwhile, 164D was one of the consensus sequences in genotype C. However, Zaini et al. showed that Q145E mutant of a C4 strain generated a mouse-virulent phenotype [31]. The substitution of T292N/K has a higher risk for nervous system complications. At this site, N292 is the consensus sequence for subtype of B1 and B2, which did not present in other genotypes. This might be the sequence basis of B1 and B2 subtypes for the elevated risk for neurovirulence than those with other subtypes or genotype. It may increase VP1-receptor binding affinity by forming an ionic bond. The residue 292 might be also responsible for receptor binding, similar to K149 of VP2 protein which interacts with one of the receptors for PSGL-1 [24].

In the case of genotype C strains, the variation N31D clearly increased the risk of nervous system infection, while I262 V decreased the risk. The N31D substitution took place in the inner of the virion structure. The replacement of I262 V mainly occurred in subtype C1–C3 and was situated at the interaction center of VP1, VP2 and VP3, and at the bottom of the canyon. This conversion may influence the hydrophobic interactions among related proteins, resulting in the loose of canyon structure. No significant change was found to be associated with neurovirulent phenotype in the C4 genotype, which mostly causes outbreaks in China, Taiwan and Japan. Cordey S et al. has identified that L97R substitution (Genbank NO.EU414333 which was included in subtype C1), could enhance the neural cell tropism in the EV71 infection [17]. In all 187 strains, there were only two clones with L97R substitution in the database, the other was Genbank NO.AF009539 which belonged into the subtype B2. Therefore, the L97R variation might not be frequent during the human EV71 infection.

Due to the limited isolates (about 180) and their geographical distribution of genotypes in this study, our findings may not represent the full picture of VP1 variations that are associated with neurological disorders. For example, we only identified 4 B1 and 8 B2 isolates that were associated with neurovirulent in Austria and US, respectively (Table 2). Other variations, which might be associated with neurovirulent phenotype, could be missed because they had not be isolated and sequenced. Other factors, for example, the fitness of virus in host would also play crucial role in the disease process. Tee et al. showed that EV71 subgenogroups have been circulating cryptically in human populations for years before causing large-scale HFMD outbreaks [32]. This would make things more complicated. On the other hand, potential biases may also arise in this study. The residue variants identified in this study needed to be further verified by large sample size with more even geographic distributions, and combined with biological functional studies in both neuron cell and animal models.

## Conclusions

In conclusion, some variations of EV71 VP1 protein associated with neurovirulent phenotype were mapped by molecular epidemiology in this study. Detection of these variations coupled with the analysis of the genotype or subtype might contribute to the prediction of clinical presentation. Further experimental data will be needed to show the significance of the results demonstrated in the present study.

## Abbreviations

EV71: Enterovirus 71; HFMD: Hand, foot and mouth disease; NS-EV71: Nervous system EV71 strains; nNS-EV71: Non-nervous system EV71 strains; S: Serine; T: Threonine; D: Aspartic acid; N: Asparagine; R: Arginine; K: Lysine; I: Isoleucine; V: Valine; L: Leucine; E: Glutamic acid; A: Alanine; P: Proline; G: Glycine; Q: Glutamine; F: Phenylalanine; H: Histidine.

## Competing interests

The authors declare that they have no competing interests.

## Authors’ contributions

BZ and LL drafted the manuscript. XW and CW analyzed the data. KH and LZ download sequences from Genbank database. MLH refined the paper. WZ carried out the design. All authors read and approved the final manuscript.

## Pre-publication history

The pre-publication history for this paper can be accessed here:

http://www.biomedcentral.com/1471-2334/14/243/prepub

## Supplementary Material

Additional file 1: Table S1 The accession number and genotype of sequences included for analysis. **Table S2.** Genotype and subgenotype distribution of EV71 strains from different countries. **Figure S1A.** Phylogenetic tree of EV71 genotype B NS, with nervous system symptoms.Subgenotypes are shown on the right, and bootstrap values (percentage of 1,000 pseudoreplicates) are shown at the nodes of the major clades. **Figure S1B.** Phylogenetic tree of EV71 genotype C NS, with nervous system symptoms.Subgenotypes are shown on the right, and bootstrap values (percentage of 1,000 pseudoreplicates) are shown at the nodes of the major clades. Click here for file
