# Supplementary material for: The variations of VP1 protein might be associated with nervous system symptoms caused by enterovirus 71 infection
Source: BMC Infect Dis. 2014 May 7;14:243. doi: 10.1186/1471-2334-14-243 (PMC4101859; doi:10.1186/1471-2334-14-243)
Supplement: Additional file 1: Table S1 — The accession number and genotype of sequences included for analysis. Table S2. Genotype and subgenotype distribution of EV71 strains from different countries. Figure S1A. Phylogenetic tree of EV71 genotype B NS, with nervous system symptoms.Subgenotypes are shown on the right, and bootstrap values (percentage of 1,000 pseudoreplicates) are shown at the nodes of the major clades. Figure S1B. Phylogenetic tree of EV71 genotype C NS, with nervous system symptoms.Subgenotypes are shown on the right, and bootstrap values (percentage of 1,000 pseudoreplicates) are shown at the nodes of the major clades. [file 1471-2334-14-243-S1.pdf]

Supplementary Table 1.

Table 1. the accession number and genotype of sequences included for analysis

NS-EV71 group	First author	reference
AB213618-C2-983-Yamagata-99-1999-Japan	Mizuta K	J Clin Microbiol. 2005;43(12):6171-5
AB550334-B3-SK-EV006-org-1997-Malaysia	Miyamura K	J Gen Virol. 2011;92(Pt 2):287-9
AB550336-B4-C7/Osaka-org-1997-Japan	Miyamura K	J Gen Virol. 2011;92(Pt 2):287-9
AF009523-B2-7962-PA-87-1987-USA	Brown BA	J Virol. 1999;73(12):9969-75
AF009525-B2-1410-CA-86-1986-USA	Brown BA	J Virol. 1999;73(12):9969-75
AF009526-B2-8102-WA-87-1987-USA	Brown BA	J Virol. 1999;73(12):9969-75
AF009527-B2-1413-CA-87-1987-USA	Brown BA	J Virol. 1999;73(12):9969-75
AF009539-B2-2219-IA-87-1987-USA	Brown BA	J Virol. 1999;73(12):9969-75
AF009542-C1-2246-NY-87-1987-USA	Brown BA	J Virol. 1999;73(12):9969-75
AF009546-C1-2264-CA-94-1994-USA	Brown BA	J Virol. 1999;73(12):9969-75
AF009549-C1-0915-MA-87-1987-USA	Brown BA	J Virol. 1999;73(12):9969-75
AF135883-B1-2604-AUS-74-1974-Japan	Brown BA	J Virol. 1999;73(12):9969-75
AF135884-B1-2605-AUS-74-1974-AUS	Brown BA	J Virol. 1999;73(12):9969-75
AF135885-B1-2608-AUS-74-1974-AUS	Brown BA	J Virol. 1999;73(12):9969-75
AF135886-B1-2609-AUS-74-1974-AUS	Brown BA	J Virol. 1999;73(12):9969-75
AF135893-B2-4224-MA-82-1982-USA	Brown BA	J Virol. 1999;73(12):9969-75
AF135895-B2-4599-OR-83-1983-USA	Brown BA	J Virol. 1999;73(12):9969-75
AF135899-B-6658-COL-94-1994-USA	Brown BA	J Virol. 1999;73(12):9969-75
AF135938-C1-2261-CA-91-1991-USA	Brown BA	J Virol. 1999;73(12):9969-75
AF135940-C1-2263-CA-94-1994-USA	Brown BA	J Virol. 1999;73(12):9969-75
AF135948-C2-2642-AUS-95-1995-AUS	Brown BA	J Virol. 1999;73(12):9969-75
AF135950-C2-2814-MO-98-1998-USA	Brown BA	J Virol. 1999;73(12):9969-75
AF136379-C2-NCKU9822-1998-taiwan	Yan JJ	J Clin Virol. 2000;17(1):13-22
AF304458-C2-Tainan/4643/98-1998-taiwan	Yan JJ	J Med Virol. 2001;65(2):331-9
AF376065-B4-SB1647/SAR/00-2000-MAL	McMinn P	J Virol. 2001;75(16):7732-8
AF376105-B3-4F/AUS/4/99-1999-AUS	McMinn P	J Virol. 2001;75(16):7732-8
AF376106-C2-5M/AUS/5/99-1999-AUS	McMinn P	J Virol. 2001;75(16):7732-8
AF376109-C2-8M/AUS/6/99-1999-AUS	McMinn P	J Virol. 2001;75(16):7732-8
AF376110-C2-9F/AUS/6/99-1999-AUS	McMinn P	J Virol. 2001;75(16):7732-8
AF376111-B4-2027/SIN/01-2001-SIN	McMinn P	J Virol. 2001;75(16):7732-8
AF376116-B3-3526/SIN/98-1998-SIN	McMinn P	J Virol. 2001;75(16):7732-8
AF376125-B4-5769/SIN/00-2000-SIN	McMinn P	J Virol. 2001;75(16):7732-8
AY125969-C3-KOR-EV71-05-2000-KOR	Cardosa MJ	Emerg Infect Dis. 2003;9(4):461-8
AY125970-C3-KOR-EV71-06-2000-KOR	Cardosa MJ	Emerg Infect Dis. 2003;9(4):461-8
AY125971-C3-KOR-EV71-07-2000-KOR	Cardosa MJ	Emerg Infect Dis. 2003;9(4):461-8
AY125972-C3-KOR-EV71-08-2000-KOR	Cardosa MJ	Emerg Infect Dis. 2003;9(4):461-8
AY125976-C3-KOR-EV71-13-2000-KOR	Cardosa MJ	Emerg Infect Dis. 2003;9(4):461-8
AY207630-C1-0808-MAA-98-1998-MAL	Herrero LJ	Arch Virol. 2003;148(7):1369-85
AY207639-B3-0245-MAA-97-1997-MAL	Herrero LJ	Arch Virol. 2003;148(7):1369-85
AY207642-B3-0899-MAA-97-1997-MAL	Herrero LJ	Arch Virol. 2003;148(7):1369-85

AY207643-B4-0898-MAA-97-MAL	Herrero LJ	Arch Virol. 2003;148(7):1369-85
AY207646-B4-0414-MAA-97-1997-MAL	Herrero LJ	Arch Virol. 2003;148(7):1369-85
AY207648-B3-0903-MAA-97-1997-MAL	Herrero LJ	Arch Virol. 2003;148(7):1369-85
DQ133458-C4-984-2004-taiwan	Li R	PLoS One. 2011;6(10):e26237.
DQ133459-C4-1235-2004-taiwan	Li R	PLoS One. 2011;6(10):e26237.
DQ341357-C2-7F/AUS/6/99-1999-AUS	Li R	PLoS One. 2011;6(10):e26237.
DQ341366-B4-SB2864/SAR/00-2000-MAL	Li R	PLoS One. 2011;6(10):e26237.
DQ341367-B3-MY821-3-SAR-97-1997-MAL	Li R	PLoS One. 2011;6(10):e26237.
ETU22521-A-Brcr-1974-USA	Li R	PLoS One. 2011;6(10):e26237.
ETU22522-B2-MS/7423/87-1987-USA	Li R	PLoS One. 2011;6(10):e26237.
EU364841-B3-26M/AUS/4/99-1999-Australia	Li R	PLoS One. 2011;6(10):e26237.
EU414333-C1-CSF-CH-06-2006-Switzerland	Cordey S	PLoS Pathog. 2012;8(7):e1002826.
EU753365-C4a-518-03F/SD/CHN/07-2007-China	Zhang Y	J Clin Virol. 2009;44(4):262-7
EU753369-C4a-521-04T/SD/CHN/07-2007-China	Zhang Y	J Clin Virol. 2009;44(4):262-7
EU753375-C4a-521-18S/SD/CHN/07-2007-China	Zhang Y	J Clin Virol. 2009;44(4):262-7
EU753397-C4a-523-05T/SD/CHN/07-2007-China	Zhang Y	J Clin Virol. 2009;44(4):262-7
EU753407-C4a-TC03F/SD/CHN/07-2007-China	Zhang Y	J Clin Virol. 2009;44(4):262-7
EU753409-C4a-TC08F/SD/CHN/07-2007-China	Zhang Y	J Clin Virol. 2009;44(4):262-7
GQ231925-B5-TW/1101/08-2008-taiwan	Chang SC	J Med Virol. 2012;84(6):931-9
GQ231926-C4-TW/1956/05-2005-taiwan	Chang SC	J Med Virol. 2012;84(6):931-9
GQ231928-C4-TW/2639/04-2004-taiwan	Chang SC	J Med Virol. 2012;84(6):931-9
GQ231929-C4-TW/2728/04-2004-taiwan	Chang SC	J Med Virol. 2012;84(6):931-9
GQ231937-C4-TW/71428/05-2005	Chang SC	J Med Virol. 2012;84(6):931-9
GQ231939-C4-TW/71595/04-2004	Chang SC	J Med Virol. 2012;84(6):931-9
GQ231941-B5-TW/96002/08-2008-taiwan	Chang SC	J Med Virol. 2012;84(6):931-9
GQ231942-B5-TW/96016/08-2008-taiwan	Chang SC	J Med Virol. 2012;84(6):931-9
GQ231943-B5-TW/96022/08-2008-taiwan	Chang SC	J Med Virol. 2012;84(6):931-9
GQ994988-C4-Anhui1-09-China-2009-China	Chang GH	Virus Res. 2010;151(1):66-73.
GQ994992-C4-Henan2-09-China-2009-China	Chang GH	Virus Res. 2010;151(1):66-73.
GU196833-C4-Henan1-09-China-2009-China	Chang GH	Virus Res. 2010;151(1):66-73.
HQ828086-C4-NBChina01-2010-China	Zhang YC	Pathol Int. 2012;62(8):565-70.
JN544418-C2-4643-TW98-1998-taiwan	Huang SW	Virology. 2012;422(1):132-43
JQ742001-C4b-AFP2001064/EV71/GX/CHN/2001-China		GenBank
JQ742002-C4b-AFP2001071/EV71/GX/CHN/2001-China		GenBank
JX244185-C4-SDLY96-2009-China	Wen HL	Virol J. 2013;10:115
JX244186-C4-SDLY107-2010-China	Wen HL	Virol J. 2013;10:115
nNS EV71 group		
AB177815-B5-2542-Yamagata-03-2003-Japan	Mizuta K	J Clin Microbiol. 2005;43(12):6171-5
AB177816-B5-2716-Yamagata-03-2003-Japan	Mizuta K	J Clin Microbiol. 2005;43(12):6171-5
AB213638-C4-1530-Yamagata-03-2003-Japan	Mizuta K	J Clin Microbiol. 2005;43(12):6171-5
AB213647-B5-2419-Yamagata-03-2003-Japan	Mizuta K	J Clin Microbiol. 2005;43(12):6171-5
AB213648-B5-2933-Yamagata-03-2003-Japan	Mizuta K	J Clin Microbiol. 2005;43(12):6171-5
AB213649-B5-2934-Yamagata-03-2003-Japan	Mizuta K	J Clin Microbiol. 2005;43(12):6171-5

AB213650-B5-2972-Yamagata-03-2003-Japan	Mizuta K	J Clin Microbiol. 2005;43(12):6171-5
AB550332-C2-1095-org-1997-japan	Miyamura K	J Gen Virol. 2011;92(Pt 2):287-9
AB550338-C4-75-Yamagata-org-2003-japan	Miyamura K	J Gen Virol. 2011;92(Pt 2):287-9
AB550340-C1-KED005-org-1997-malaysia	Miyamura K	J Gen Virol. 2011;92(Pt 2):287-9
AF119796-C2-TW/2086/98-1998-taiwan	Shih SR	Virus Res. 2000;68(2):127-36.
AF304457-C2-Tainan/5746/98-1998-Taiwan	Yan JJ	J Med Virol. 2001;65(2):331-9
AF304459-C2-Tainan/6092/98-1998-taiwan	Yan JJ	J Med Virol. 2001;65(2):331-9
AF352027-B4-Enterovirus_5666/sin/002209-2000-SIN	Singh S	Microbiol Immunol. 2002;46(11):801-8
AF376070-B4-CN0942/SAR/00-2000-MAL	McMinn P	J Virol. 2001;75(16):7732-8
AF376071-B4-CN9502/SAR/00-2000-MAL	McMinn P	J Virol. 2001;75(16):7732-8
AF376078-B3-MY860-3/SAR/97-1997-MAL	McMinn P	J Virol. 2001;75(16):7732-8
AF376079-C1-S10822/SAR/98-1998-MAL	McMinn P	J Virol. 2001;75(16):7732-8
AF376081-C1-S11051/SAR/98-1998-MAL	McMinn P	J Virol. 2001;75(16):7732-8
AF376084-B4-S21082/SAR/00-2000-MAL	McMinn P	J Virol. 2001;75(16):7732-8
AF376088-B3-10M/AUS/6/99-1999-AUS	McMinn P	J Virol. 2001;75(16):7732-8
AF376120-C1-4575/SIN/98-1998-SIN	McMinn P	J Virol. 2001;75(16):7732-8
AF376122-B4-5536/SIN/00-2000-SIN	McMinn P	J Virol. 2001;75(16):7732-8
AM396584-C2-ENT/PM/SHA52-1997-MAL	Chan YF	BMC Microbiol.2006;6:74.
AM396585-C2-ENT/PM/SHA71-1997-MAL	Chan YF	BMC Microbiol.2006;6:74.
AM396586-B3-EV71/SAR/SHA66-1997-MAL	Chan YF	BMC Microbiol.2006;6:74.
AM396588-B3-EV71/SAR/SHA63-1997-MAL	Chan YF	BMC Microbiol.2006;6:74.
AY125966-C3-KOR-EV71-01-2000-KOR	Cardosa MJ	Emerg Infect Dis. 2003;9(4):461-8
AY125967-C3-KOR-EV71-02-2000-KOR	Cardosa MJ	Emerg Infect Dis. 2003;9(4):461-8
AY125968-C3-KOR-EV71-3-2000-KOR	Cardosa MJ	Emerg Infect Dis. 2003;9(4):461-8
AY125973-C3-KOR-EV71-09-2000-KOR	Cardosa MJ	Emerg Infect Dis. 2003;9(4):461-8
AY125974-C3-KOR-EV71-10-2000-KOR	Cardosa MJ	Emerg Infect Dis. 2003;9(4):461-8
AY125975-C3-KOR-EV71-11-2000-KOR	Cardosa MJ	Emerg Infect Dis. 2003;9(4):461-8
AY207611-C2-03907-MAA-97-1997-MAL	Herrero LJ	Arch Virol. 2003;148(7):1369-85
AY207612-C1-03784-MAA-97-1997-MAL	Herrero LJ	Arch Virol. 2003;148(7):1369-85
AY207613-B4-03300-MAA-97-1997-MAL	Herrero LJ	Arch Virol. 2003;148(7):1369-85
AY207614-B3-0870-MAA-97-1997-MAL	Herrero LJ	Arch Virol. 2003;148(7):1369-85
AY207615-C2-03750-MAA-97-1997-MAL	Herrero LJ	Arch Virol. 2003;148(7):1369-85
AY207616-B3-0473-MAA-97-1997-MAL	Herrero LJ	Arch Virol. 2003;148(7):1369-85
AY207617-B4-0778-MAA-00-1997-MAL	Herrero LJ	Arch Virol. 2003;148(7):1369-85
AY207618-C1-0807-MAA-00-2000-MAL	Herrero LJ	Arch Virol. 2003;148(7):1369-85
AY207619-C1-0836-MAA-00-2000-MAL	Herrero LJ	Arch Virol. 2003;148(7):1369-85
AY207620-C1-0915-MAA-00-2000-MAL	Herrero LJ	Arch Virol. 2003;148(7):1369-85
AY207621-C1-0937-MAA-00-2000-MAL	Herrero LJ	Arch Virol. 2003;148(7):1369-85
AY207622-C1-0948-MAA-00-2000-MAL	Herrero LJ	Arch Virol. 2003;148(7):1369-85
AY207623-B4-0042-MAA-00-1997-MAL	Herrero LJ	Arch Virol. 2003;148(7):1369-85
AY207624-B4-0066-MAA-00-2000-MAL	Herrero LJ	Arch Virol. 2003;148(7):1369-85
AY207625-C1-0113-MAA-00-2000-MAL	Herrero LJ	Arch Virol. 2003;148(7):1369-85
AY207626-C1-0431-MAA-00-1997-MAL	Herrero LJ	Arch Virol. 2003;148(7):1369-85
AY207627-B4-0467-MAA-00-2000-MAL	Herrero LJ	Arch Virol. 2003;148(7):1369-85

AY207628-B4-03907-MAA-97-1997-MAL	Herrero LJ	Arch Virol. 2003;148(7):1369-85
AY207629-C1-0838-MAA-99-1999-MAL	Herrero LJ	Arch Virol. 2003;148(7):1369-85
AY207631-C1-0557-MAA-98-1998-MAL	Herrero LJ	Arch Virol. 2003;148(7):1369-85
AY207632-C1-0832-MAA-00-2000-MAL	Herrero LJ	Arch Virol. 2003;148(7):1369-85
AY207633-B5-0815-MAA-00-2000-MAL	Herrero LJ	Arch Virol. 2003;148(7):1369-85
AY207634-C1-0774-MAA-00-2000-MAL	Herrero LJ	Arch Virol. 2003;148(7):1369-85
AY207635-C1-05716-MAA-00-1997-MAL	Herrero LJ	Arch Virol. 2003;148(7):1369-85
AY207636-B3-04716-MAA-97-1997-MAL	Herrero LJ	Arch Virol. 2003;148(7):1369-85
AY207637-B4-0343-MAA-97-1997-MAL	Herrero LJ	Arch Virol. 2003;148(7):1369-85
AY207638-C1-0283-MAA-97-1997-MAL	Herrero LJ	Arch Virol. 2003;148(7):1369-85
AY207640-B4-0175-MAA-97-1997-MAL	Herrero LJ	Arch Virol. 2003;148(7):1369-85
AY207641-B3-0036-MAA-97-1997-MAL	Herrero LJ	Arch Virol. 2003;148(7):1369-85
AY207644-B3-0897-MAA-97-1997-MAL	Herrero LJ	Arch Virol. 2003;148(7):1369-85
AY207645-B3-0884-MAA-97-1997-MAL	Herrero LJ	Arch Virol. 2003;148(7):1369-85
AY207647-B4-0128-MAA-97-1997-MAL	Herrero LJ	Arch Virol. 2003;148(7):1369-85
AY207650-B4-0919-MAA-99-1999-MAL	Herrero LJ	Arch Virol. 2003;148(7):1369-85
DQ341354-B3-3799/SIN/98-1998-SIN	McMinn P	J Virol. 2001;75(16):7732-8
DQ341358-C1-S40221/SAR/00-2000-MAL	McMinn P	J Virol. 2001;75(16):7732-8
DQ341359-C1-S10862/SAR/98-1998-MAL	McMinn P	J Virol. 2001;75(16):7732-8
DQ341361-C1-1M/AUS/12/00-2000-AUS	McMinn P	J Virol. 2001;75(16):7732-8
DQ341364-B5-5511/SIN/00-2000-SIN	McMinn P	J Virol. 2001;75(16):7732-8
DQ452074-C1-804/NO/03-healthy_child-2003-Norway	McMinn P	J Virol. 2001;75(16):7732-8
EU753366-C4a-519-02F/SD/CHN/07-2007-China	Zhang Y	J Clin Virol. 2009;44(4):262-7
EU753372-C4a-522-04T/SD/CHN/07-2007-China	Zhang Y	J Clin Virol. 2009;44(4):262-7
EU753378-C4a-521-23F/SD/CHN/07-2007-China	Zhang Y	J Clin Virol. 2009;44(4):262-7
EU753379-C4a-521-25F/SD/CHN/07-2007-China	Zhang Y	J Clin Virol. 2009;44(4):262-7
EU753384-C4a-522-04T/SD/CHN/07-2007-China	Zhang Y	J Clin Virol. 2009;44(4):262-7
EU753398-C4a-523-07T/SD/CHN/07-2007-China	Zhang Y	J Clin Virol. 2009;44(4):262-7
EU753417-C4a-TC23F/SD/CHN/07-2007-China	Zhang Y	J Clin Virol. 2009;44(4):262-7
EU812515-C4-FY23-2008-China	Wang LC	Virol Sin. 2010;25(2):98-106
FJ439769-C4-Fuyang-0805-2008-China	Wu Z	J Virol Methods. 2009;159(2):233-8
FJ606447-C4-BJ08-Z004-3-2008-China	Ding NZ	Virus Res. 2009;145(1):157-61.
FJ606448-C4-BJ08-Z011-4-2008-China	Ding NZ	Virus Res. 2009;145(1):157-61.
FJ607334-C4-1/SHENZHEN/08/China/HFMD/2008-China	Yu Z	Arch Virol. 2013;158(5):1071-7
FJ607335-C4-4/SHENZHEN/08/China/HFMD/2008-China		Genbank
FJ607336-C4-28/SHENZHEN/08/China/HFMD/2008-China		Genbank
FJ828519-C4-BJ08-2008-China	Mao Q	PLoS One. 2012;7(9):e46043
GQ121418-C4a-EV71/Fuyang.Anhui.P.R.C/17.08/5-2008-China	Zhang Y	Virol J. 2010;7:94.
GQ231927-C4-TW/2429/04-2004-taiwan	Chang SC	J Med Virol. 2012;84(6):931-9
GQ231930-C4-TW/2815/04-2004-taiwan	Chang SC	J Med Virol. 2012;84(6):931-9
GQ231931-C4-TW/2824/04-2004-taiwan	Chang SC	J Med Virol. 2012;84(6):931-9
GQ231932-C4-TW/2871/04-2004-taiwan	Chang SC	J Med Virol. 2012;84(6):931-9
GQ231933-C4-TW/70516/08-2008-taiwan	Chang SC	J Med Virol. 2012;84(6):931-9
GQ231935-B5-TW/70886/08-2008-taiwan	Chang SC	J Med Virol. 2012;84(6):931-9

GQ231936-B5-TW/70902/08-2008-taiwan	Chang SC	J Med Virol. 2012;84(6):931-9
GQ231940-C4-TW/72232/04-2004-taiwan	Chang SC	J Med Virol. 2012;84(6):931-9
GQ994989-C4-Chongqing1-09-China-2009-China	Chang GH	Virus Res. 2010;151(1):66-73.
GQ994990-C4-Chongqing2-09-China-2009-China	Chang GH	Virus Res. 2010;151(1):66-73.
GQ994991-C4-Chongqing3-09-China-2009-China	Chang GH	Virus Res. 2010;151(1):66-73.
HM037793-C4-GD08-EV71-039-2008-China	Sun LM	Jpn J Infect Dis. 2011;64(1):13-8
HM037797-C4-GD08-EV71-194-2008-China	Sun LM	Jpn J Infect Dis. 2011;64(1):13-8
HM037798-C4-GD08-EV71-274-2008-China	Sun LM	Jpn J Infect Dis. 2011;64(1):13-8
HM037799-C4-GD08-EV71-274-2008-China	Sun LM	Jpn J Infect Dis. 2011;64(1):13-8
HM037802-C4-GD08-EV71-495-2008-China	Sun LM	Jpn J Infect Dis. 2011;64(1):13-8
HM037806-C4-GD08-EV71-614-2008-China	Sun LM	Jpn J Infect Dis. 2011;64(1):13-8
HQ129932-C4-BJ06-SJS06-healthy_child-2006-China	Li R	PLoS One. 2011;6(10):e26237.
HQ882182-C4-FY0805-2008-China	Liu J	Virol J. 2011 Oct 27;8:483.
JX025561-C4-FY7VP5/AH/CHN/2008-China		Genbank
JX244182-C4-SDLY1-2008-China	Wen HL	Virol J. 2013;10:115
JX244183-C4-SDLY11-2008-China	Wen HL	Virol J. 2013;10:115
JX244184-C4-SDLY48-2008-China	Wen HL	Virol J. 2013;10:115

Supplementary Table 2

Table 2. Genotype and subgenotype distribution of EV71 strains from different countries

Country	NS or nNS	A	B					C			
			B1	B2	B3	B4	B5	C1	C2	C3	C4
Malaysia	NS				5	4		1			
	nNS				9	13	1	19	4		
Austria	NS		4		2				5		
	nNS				1			1			
Singapore	NS				1	2					
	nNS				1	2	1	1			
USA	NS	1		8				5	1		
Taiwan	NS						4		3		7
	nNS						2		3		6
Japan	NS					1		1			
	nNS						6	1			2
China	NS										14
	nNS										31
Korea	NS									5	
	nNS									6	
Switzerland	NS							1			
Norway	nNS							1			

Note: AF135899 strain is not included because it belongs to none of the subgenotypes.

Supplementary Fig.1 A

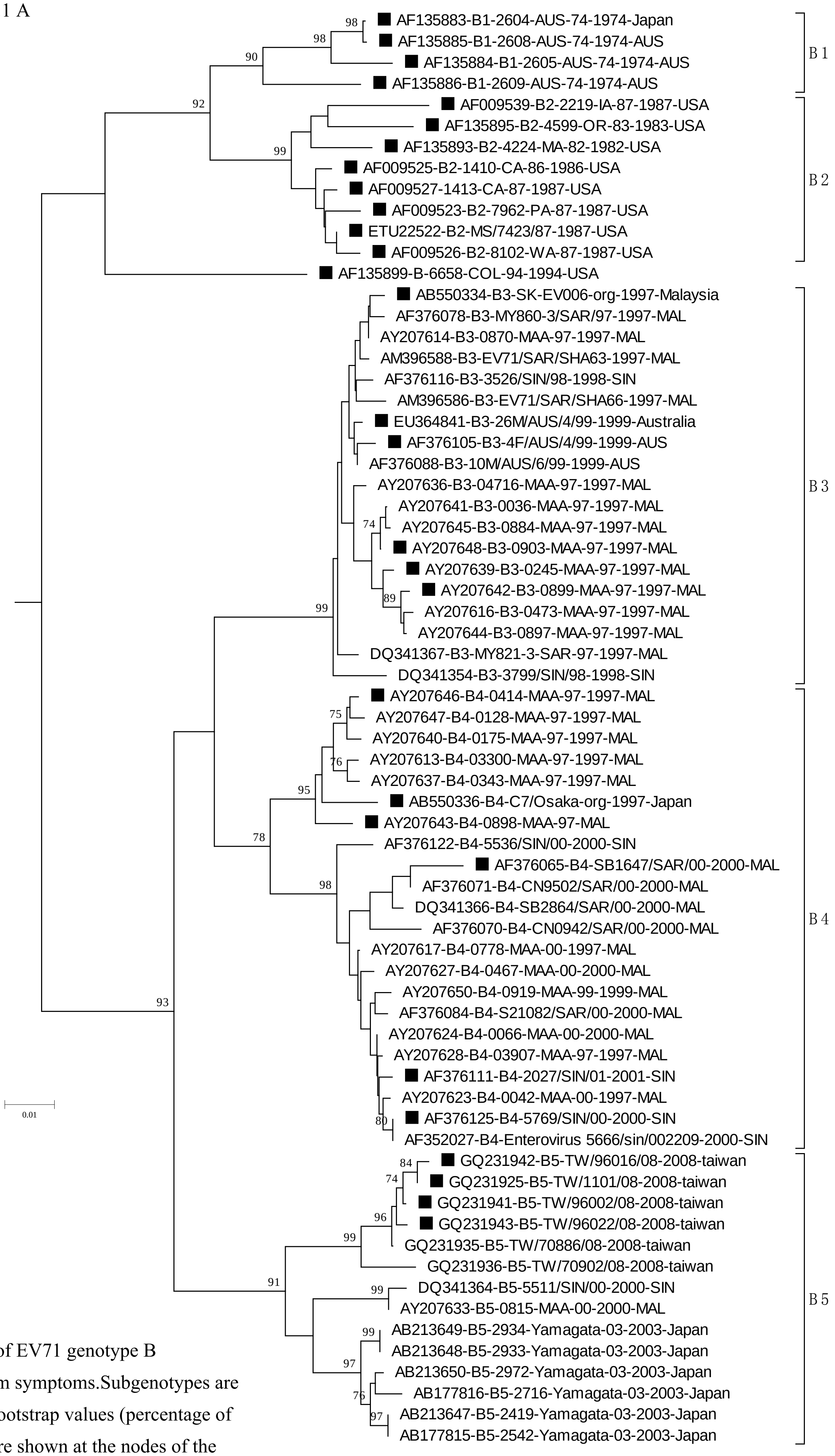

Fig.1A phylogenetic tree of EV71 genotype B

■ NS, with nervous system symptoms.Subgenotypes are shown on the right, and bootstrap values (percentage of 1,000 pseudoreplicates) are shown at the nodes of the major clades.

Supplementary Fig.1 B

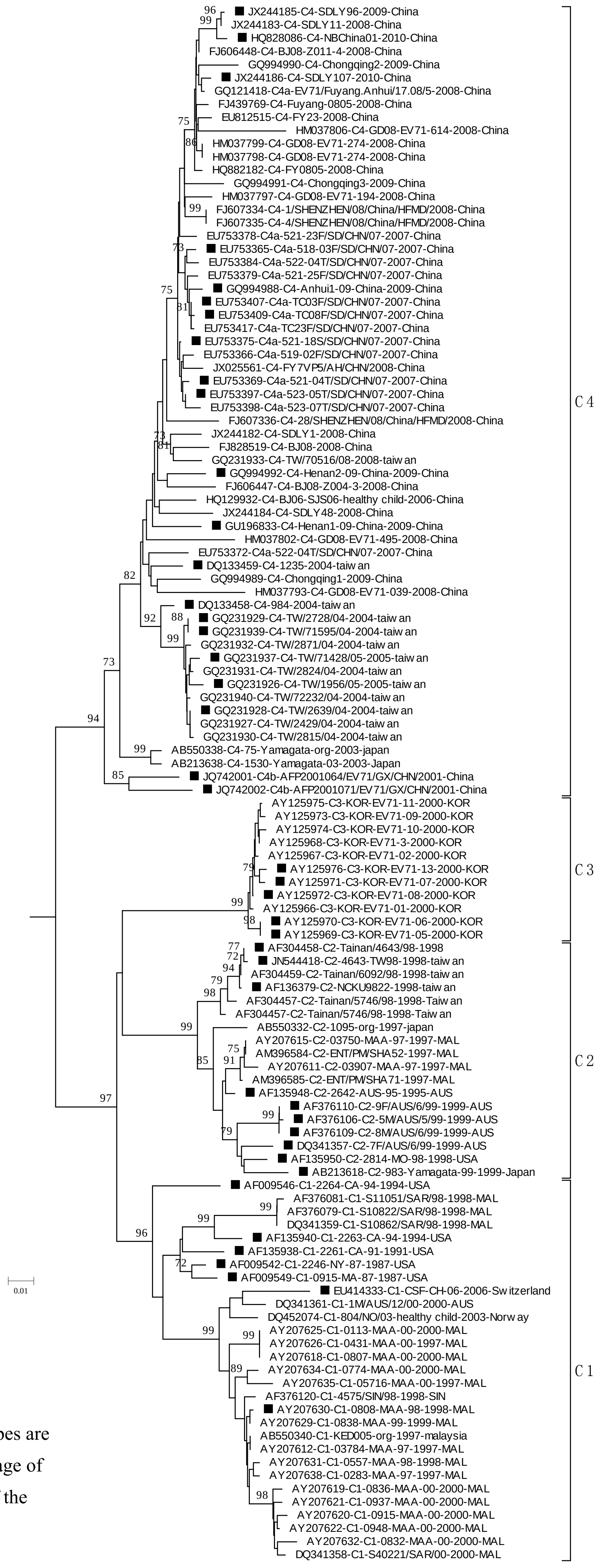

Fig.1B phylogenetic tree of EV71 genotype C

■ NS, with nervous system symptoms.Subgenotypes are shown on the right, and bootstrap values (percentage of 1,000 pseudoreplicates) are shown at the nodes of the major clades.
